# Family planning utilization and factors associated among women receiving abortion services in health facilities of central zone towns of Tigray, Northern Ethiopia: a cross sectional Study

**DOI:** 10.1186/s12905-018-0582-4

**Published:** 2018-06-05

**Authors:** Goshu Hagos, Gurmesa Tura, Gizienesh Kahsay, Kebede Haile, Teklit Grum, Tsige Araya

**Affiliations:** 1 School of Public Health, College of Health Sciences, Aksum University, Axum, Ethiopia; 2 School of Public Health, College of Health Sciences, Jimma University, Jimma, Ethiopia; 3 Department of Midwifery, College of Health Sciences, Mekelle University, Mekelle, Ethiopia

**Keywords:** Post abortion contraceptive use, Women, Health facilities, Ethiopia

## Abstract

**Background:**

Abortion remains among the leading causes of maternal death worldwide. Post-abortion contraception is significantly effective in preventing unintended pregnancy and abortion if provided before women leave the health facilty. However, the status of post-abortion family planning (PAFP) utilization and the contributing factors are not well studied in Tigray region. So, we conduct study aimed on family planning utilization and factors associated with it among women receiving abortion services.

**Methods:**

A facility based cross-sectional study design was conducted among women receiving abortion services in central zone of Tigray from December 2015to February 2016 using a total of 416 sample size. Women who came for abortion services were selected using systematic random sampling technique.. The data were collected using a pre-tested interviewer administered questionnair. Data were coded and entered in to Epi info 7 and then exported to SPSS for analysis. Descriptive statisticslike frequencies and mean were computed to display the results. Both Bivariable and multivariable logistic regression was used in the analysis. Variables statistically significant at *p* < 0.05 in the bivariable analysis were checked in multivariable logistic regration to identify independently associated factors. Then variables which were significantly associated with post abortion family planning utilization at *p*-value < 0.05 in the multivariable analysis were declared as significantly associated factors.

**Results:**

A total of 409 abortion clients were interviewed in this study with 98.3% of response rate. Majority 290 (70.9%) of study participants utilized contracepives after abortion. Type of health facility, the decision maker on timing of having child, knowledge that pregnancy can happen soon after abortion and husband’s opposition towards contraceptives were significantly associated with Post-abortion family planning ustilization.

**Conclusions:**

About one-third of abortion women failed to receive contraceptive before leaving the facility. Private facilities should strengthen utilization of contraceptives on post abortion care service. Health providers should provide counseling on timing of fertility-return following abortion before women left the facility once they receive abortion care. Women empowerment through enhancing community’s awareness focusing on own decision making in the family planning utilization including the partner should be strengthened.

## Background

Globally, there were an estimated 289,000 maternal deaths in 2013. The sub-Saharan Africa region alone accounted for 62% (179,000) of global deaths followed by Southern Asia at 24% (69,000) [1]. In 2013, 43,684 women lost their lives as a result of complications from abortion worldwide. Abortion remains among the leading causes of maternal death worldwide [2].

Unsafe abortion continues to be serious public health problem particularly in developing countries especially where abortion laws are restrictive [3]. Ethiopia is one of the countries with highest maternal mortality rate in the world where unsafe abortion remains to be among the top causes of Maternal Mortality (MM) which contributes 10% [4].

Family planning (FP) is a major primary prevention strategy for unwanted pregnancies at which contraceptive use reduces about 230 million births every year worldwide [5].

The World Health Organization (WHO) recommends a six month inter-pregnancy interval following abortion to ensure better maternal health [6]. Following an induced or spontaneous abortion, all women should receive counseling and contraception services to prevent unintended pregnancies in their future. Abortion service delivery sites should be p provided in a place where women can decide freely based on their interest without any interferences on contraceptive method of choice [7].

Providing post abortion contraceptive improves family planning acceptance which breaks the cycle of having unwanted pregnancy. Since ovulation can occur soon (as early as 10 days) after the abortion before seeing her menses. The women should be provided with family planning services at the site of care right away after the abortion process is over before leaving the health-care facility [8].

Despite the efforts have done in improving maternal health, post abortion family planning use is not well addressed. Therefore, this study was aimed to assess post-abortion FP utilization and identify factors affecting post-abortion contraceptive use.

## Methods

### Study setting

Facility based cross-sectional study design was used. The study was conducted in central zone of Tigray region, northern Ethiopia from December 05, 2015 to February 05, 2016. Central zone of Tigray is located at a distance of 1245 km far from Addis Ababa. The population size of this zone was estimated 1,131,697. Out of these 576,367 (51%) were females and 556,330 males (49%). The zone includes 9 district woredas and 3 administrative towns. Health facilities that are providing abortion services in the 3 administrative towns (Aksum, Adwa and Abi-Adi) are 3 general hospitals, 5 health centers (HC), 2 private clinicsfor-profit and 1 Reproductive health based non-governmental organization (NGO) [9].

Source population.

All women who received abortion care services in central zone of Tigray during the data collection period.

### Inclusion criteria

All women who received abortion care services in the selected health facilities during the data collection were included in the study.

### Exclusion criteria

Clients, who could not talk or listen and have mental problem were excluded from the study.

### Sample size determination

**S**ample size was calculated using the single population proportion formula based on the following assumptions, 95% confidence interval with a 5% margin error, the expected proportion of post abortion contraceptive use to be 56.5% (*p* = 0.565) [10]. The final sample size is therefore determined by adding a 10% non-response rate.

$$ {\displaystyle \begin{array}{c}\mathrm{Formula}\;\left(\mathrm{N}\right)=\frac{{\left(\raisebox{1ex}{$ Za$}\!\left/ \!\raisebox{-1ex}{$2$}\right.\right)}^2p\left(1-p\right)}{w^2}\kern1.92em \raisebox{1ex}{$ Za$}\!\left/ \!\raisebox{-1ex}{$2$}\right.=1.96\kern0.24em \mathrm{at}\;\mathrm{CI}\;\mathrm{of}\;95\%\\ {}\left(\mathrm{N}\right)={(1.96)}^2\;\left(0.565\times 0.435\right)/{(0.05)}^2\kern0.24em \mathrm{N}=378\end{array}} $$

By adding 10% non response rate, the final required sample size was estimated as = 416

### Sampling procedure

All health facilities providing abortion care services in central zone towns of Tigray, (3 public hospitals, 5 HCs, 1 NGO, and 2 private for profit clinics) were included in the study.

**Fig. 1:**
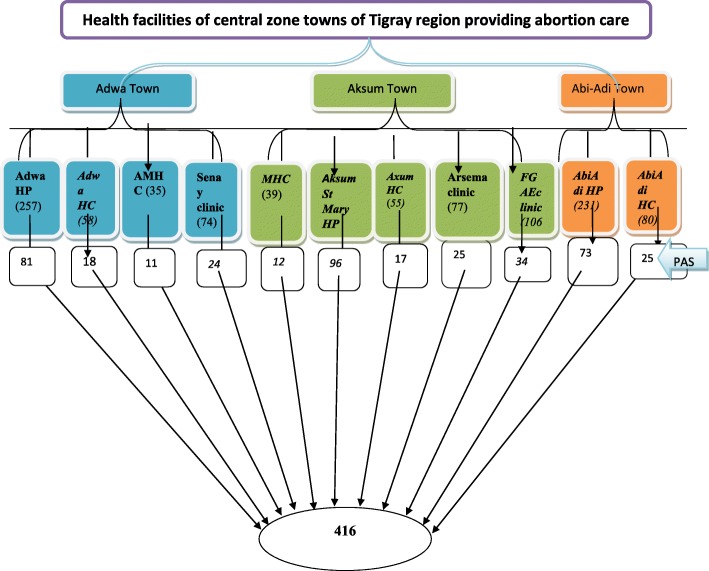
Schematic presentation of sampling procedure of proportional allocation to size in selected health facilities of central zone of Tigray region. 2015

The average number of women coming for abortion service was estimated based on recent quarter report of client load in each health facilities. The number of study subjects sampled between health facilities were allocated using population proportion to size. Systematic random sampling technique was used to select the study units (Fig. 1).

### Data collection tools and procedures

The data were collected using structured and pretested questionnaire by interviewer administered technique. The questionnaires were adapted from reviewing previous relevant literatures and the Ethiopian DHS 2011 by considering the national and local context of the study subjects [11]. The questionnaire was prepared in English and then translated in to local Tigrigna language. The Tigrigna version was again translated back to English version to check for consistency of meaning. Based on the findings of the pre-test, modification and development of the tool was made.

Clients exit interview was conducted at a point where the service users were ready to be discharged. Eleven female diploma nurses and three degree nurses with bachelor of sciences were hired as data collectors and supervisors respectively during the data collection process. Tarining for data collector along with supervisors was given for two days by the principal investigator related to the sampling techniques, quality control, completeness and ethics of the data collection process. Supervisors were on-top to monitor the whole data collection process.

### Data processing and analysis

Data were entered using Epi-info version 3.5.4 and was exported to SPSS version 20 for cleaning & further analysis. Descriptive statistics like: frequency, proportion, mean, and median were computed and presented using tables and texts. Both Bivariable and multivariable analysis was done to check the association between the independent and dependent variable. Collinearity was chaceked using violence inflation factor (VIF). All factors with p–value < 0.05 in the bivariable analysis were included in multivariable logistic regressions to control confounding effect. Adjusted Odds Ratio (AOR) with 95% confidence intervals (95% CI) was calculated to measure the strength of association. Variables at p–value < 0.05 in multivariable logistic regressions have been considered as significant association with the outcome.

### Operational definitions

#### Attitude of women

Classified as favorable and unfavorable attitude. Women were considered to have unfavourable attitude for a given item they scores greater than or equal to the statically cut-off (total mean score) and considered to have favourable attitude if thay score less than the statistically cut-off point (total mean score).

#### Knowledge of women

PAFP related Knowledge variables were analyzed separetly.

### Data quality assurance

Training was provided by the principal investigator to the data collectors and supervisors. The study used proper designed data collection tool & pre-tested questionnair. Supervisors and Principal investigator were checked the completeness and correctness of each questionnaire on daily basis.

## Results

### Socio-demographic characteristics of respondents

Out of 416 women estimated in sample size, atotal of 409 abortion clients were interviwed in this study with response rate of 98.3%. The mean age of study particpants was 24.2 (SD ±6.11). About two-third of the respondents 249 (60.9%) were in the age group of 15–24 years. Majority of the respondents 329 (80.4%) were served in public health facility and urban residents 295 (72%). Three hundred seventy four (91.4%) of the study participants belongs to Tigray ethinicity. The study participants with educational status of secondary school (9–12 grade) were 136 (33.3%). Regarding to marital status and occupation, 188 (46%) were single and 140 (34.2%) were house wife respectively. The median monthly income was $ 26. Most of respondents 172 (42.1%) were in the income category of $15–26 per month. The majority of study subjects 360 (88%) were Orthodox Christian followers. Concerning to family size, only 64(15.6%) of the study subjects were live with 5 or above family size (Table 1).

**Table 1:** Socio-demographic characterstics of the respondents, central zone of Tigray, North Ethiopia, 2015

Variables	Frequency (N)	Percent (%)
Age		
15–24	249	60.9
25–34	119	29.1
35+	41	10
Residence		
Urban	295	72.1
Rural	114	27.9
Ethnicity		
Tigray	374	91.4
Amhara	30	7.3
Others	5	1.2
Education		
Can not read and write	78	19.1
Can only read and write	33	8.1
Primary school [1–8]	94	23.0
Secondary school [9–12]	136	33.3
College and higher	68	16.6
Occupation		
House wife	140	34.2
Student	129	31.5
Employed	74	18.1
Un employed	26	6.4
Commercial sex worker	21	5.1
Trader	19	4.6
Marital status		
Married or living together	178	43.5
Single	188	46.0
Divorced and Widowed	43	11.0
Monthly income		
≤ $15	167	40.8
$16–26	172	42.1
$27–55	51	12.5
> $55	19	4.6
Religion		
Orthodox	360	88.0
Muslim	35	8.6
Others (Protestant & Catholic)	14	3.4
Family size		
1–4	345	84.4
5 and above	64	15.6

Almost all 400(97.8%) of the study subjects were counseled for post abortion family planning and 216 (52.8%) of them were counseled during the procedure. Even though almost all were counseled, 290 (70.9%) of them were received the contraceptive after abortion service. Regarding to type of health facility served, 329(80.4%) of the contraceptive users were served in public health facilities (Table 2).

**Table 2:** Post-abortion contraceptive use and facility related variables, central zone of Tigray, North Ethiopia, 2015 (*n* = 409)

Variables	Frequency (N)	Percent (%)
Counseled for FP		
Yes	400	97.8
No	9	2.2
When was the FP Counseling provided		
Before the procedure	216	52.8
During the procedure	42	10.3
After the procedure	151	36.9
Received contraception after abortion		
Yes	290	70.9
No	119	29.1
Room (place) received the contraceptive (*n* = 290)		
At the abortion room	221	76.20
At FP room	65	22.4
other rooms (OPD/ Gynecology)	4	1.4
Type of health facility served (n = 409)		
Public	329	80.4
Private	47	11.5
NGO	33	8.1

Bivariable logistic regration was used to determine the association of each independent variables with post abortion contraceptive use with out controlling the effect of any other variables. Variables which were significantly associated with post abortion FP use in bivariable logistic regression were; health facility type, pregnancy history, abortion history, plan before the index pregnancy, the index pregnancy planning, the main decider of when to have child, knowledge on a women can get pregnant sooner after abortion, how soon can a women get pregnant again after abortion, husband’s attitude towards contraceptive use and the time of counselling conducted..

From the variables associated with PAFP in bivariable logistic regression; type of health facilities, the decider of when to have child, know a women can get pregnant sooner after abortion, and husband’s attitude towards contraception were statistically significant with the PAFP in the multivariable logistic regression analysis.

Multivariable logistic regression analysis identified type of health facility served has strongest association with PAFP. Individuals served in NGO were 6.7 times more likely to receive contraceptives than individuals served in public facilities AOR = 6.668 [95% CI; 1.418, 31.361] where as, abortion clients served in private for profit facilities were 72.4% less likely to utilize as compared to the public facilities AOR = 0.276 [95% CI; 0.127, 0.601].

In this study, the main decision maker on when to have child was also found to be significant factor for receiving PAFP. The odds of receiving PAFP in clients whom their husband were the main deciders were 85% less likely to receiving contraception as compared to the women who make decisions themselves AOR = 0.149 [95% CI; 0.034, 0.650]. Participants who had opposition from their husband to receiving contraception were about 77.7% less likely to utilize PAFP before leaving the facilty than compared to the women who are supported by their husbands AOR = 0.223 [95% CI; 0.103, 0.482]. Those who were uncertain of their husbands’ attitude towards contraceptive were also 66% less likely to receive FP AOR = 0.3403 [95% CI; 0.160, 0.722].

Utilizing of PAFP was also increased with knowledge of study participants on knowing fertility returns sooner which was also significantly associated. Study participants who knows that could get pregnant again sooner after abortion (10-14 days) were observed to utilize PAFP 2 times than those who do not know AOR = 2.188 (1.105, 4.334) (Table 3).

**Table 3:** Multivariable analysis of factors associated with Post-abortion contraceptive use, central zone of health facilities, Tigray, North Ethiopia, 2015 (n = 409)

Variables		PAFP utilization		COR 95% CI	AOR 95% CI
		No Frequency /%	Yes Frequency /%		
Type of Health facility	Public	88(73.9)	241(83.1)	1	1
	Private	29(24.4)	18(6.2)	0.23(0.12,0.43)*	0.28(0.13, 0.6)*
	NGO	2(1.7)	31(10.7)	5.66(1.33, 24.14)*	6.67(1.4,31.36)*
Had history of Pregnancy	Yes	81(68.1)	151(52.1)	0.51(0.33, 0.8)*	0.66 (0.33, 1.35)
	No	38(31.9)	139(47.9)	1	1
Had history of previous abortion	Yes	51(42.9)	72(24.8)	2.27(1.45, 3.56)*	0.58 (0.29, 1.16)
	No	68(57.1)	218(75.2)	1	1
Pregnancy plan before the index Pregnancy	To get pregnant	38(31.9)	55(19.0)	1	1
	Later	76(63.9)	210(72.4)	1.91(1.17, 3.12)*	1.25 (0.08, 19.28)
	Not at all	5(4.2)	25(8.6)	3.46(1.21, 9.83)*	3.14 (0.18, 54.95)
Planning of the index pregnancy	Yes	40(33.6)	57(19.7)	1	1
	No	79(66.4)	233(80.3)	2.07(1.28, 3.34)*	2.54 (0.17, 39.13)
Decision maker on FP use	Wife	4(3.4)	19(6.6)	1	1
	Husband	21(17.6)	12(4.1)	0.12(0.33, 0.44)*	0.15(0.03, 0.65)*
	Both	47(39.5)	190(65.5)	0.851(0.28, 2.72)	0.83(0.23, 3.04)
	I don’t know	47(39.5)	69(23.8)	0.31(0.01, 0.97)*	0.27(0.07, 1.03)
Knowing on time getting pregnant again	Yes	43(36.1)	172(59.3)	2.58(1.66, 4.01)*	2.19(1.1, 4.3)*
	No	76(63.9)	118(40.7)	1	1
Knowledge on how soon fertility returns and could get pregnant again	within 10–14 days	18(15.1)	92(31.7)	1	1
	After 3–4 wks	25(21.0)	80(27.6)	0.63(0.32, 1.23)	0.79(0.37, 1.7)
	don ‘t know	76(63.9)	118(40.7)	0.3(0.17, 0.54)*	1.91(0.1, 4.3)
Husbands’ attitude on contraceptive use	Approve	30(25.2)	125(43.1)	1	1
	Disapprove	42(35.3)	46(15.9)	0.26(0.19, 0.47)*	0.34 (0.160, 0.72)*
	Don’t know	47(39.5)	119(41.0)	0.61(0.36, 1.02)	0.22(0.10, 0.48)*
Time of FP counseling provided	Before procedure	43(36.1)	173(59.7)	2.9(1.81, 4.59)*	1
	During procedure	13(10.9)	29(10.0)	1.6(0.77, 3.3)	1.6 (0.9, 2.9)
	After the procedure	63(52.9)	88(30.3)	1	1.8(0.71, 4.57)

*→ *P*-value< 0.05

## Discussion

We conducted a study aimed on determining the proportion of women who used PAFP among those who received abortion care and possible factors that may associate with the PAFP service utilization. In this study majority of the participants, 290 (70.9%), received post abortion contraceptives before they leave the facility which is lower than study done in Brazil (97.4%), Nepal (83%), AddisAbeba (86%) and Tanzania (89%) [11–14] respectively.

The possible disparity may be related to difference in knowledge on contraceptive since all res pondents in Brazilstudy and 80% respondents in Nepal study had good knowledge on contraceptives compared to this study. Higher educational level (about 80%) of Addis Abeba study respondents might be the reason for higher use of PAFP as compared to this study. The Tanzania study design was a cohort study which could be a reason for the discrepancy from this current study. The other possible reasons might be due to low counseling skills and cultural difference.

However, it is higher as compared to study conducted in Gurage (56.5%) and Dessie town (47.5%) of Ethiopia at which only about half left the health facility with contraceptives [15, 16]. The disparity could be due to study year difference as there is health care improvement through time. This result is observed to be consistent with a study conducted in Pakistan (72.9%) [17].

Type of health facility has been significantly associated with PAFP use which is consistent with study conducted in Ethiopia AOR = 0.01 [95% CI; 0.001, 0.08]. Less than half of clients (38.29%) out of respondents served in private facilities did not received contraceptives compared to abortion women served in NGO. In this study PAFP utilization is higher compared to other study conducted in Ethiopia at which only 13% have received post abortion contraceptions [16]. Facility type that provide abortion services has great association with receiving PAFP.Clients who received abortion care in private facilities had less likely to utilize post abortion contraceptives [13].

This could be due to private facilities primarilywork for-profit and pocket fee compared to the Public or NGO who serve FP freely. This calls for policies to strengthening and institutionalization of postabortion family planning services in private health sector over time.

In this study about half 215(52.6%) of the study subjects reported that they could get pregnant immidately or early after abortion if they did not receive contraceptives. Out of which only half (26.9%) of respondents mentioned the correct time. I.e fertility could return after 10-14 days following abortion. This result is lower than studies conducted in Addis Abeba (73%) and in Egypt (56.7%) [18, 19]. This could be due to the participants in Addis Abeba and Egypt were urban residents and with higher educational level. However, this result is higher than the study done in Dessie (34%) [16] which seem improved as compared to the past evidences studied some years before the current study. This shows respondents are leaving the facility with out knowing fertility return time.

This is inline with studies conducted in Afghanistan and Russia documented at wich PAFP utilization is associated with counseling skills of service providers. High counseling skills of providers improves FP use [20, 21]. This implies that there is a need to improve providers’ FP counselling skills in general and mainly on fertility return time after abortion. The good-opportunity to prevent repeated un-intended pregnancy and perhaps repeated abortions is lesser ifclients with abortion care are receiving an effective counseling on PAFP use.

In this study women who had opposition attitude from their husband hinders to receive contraception. Women who had males opposition were about 77.7% less likely to utilize FP before leaving the facilty than their counter parts AOR = 0.223 [95% CI; 0.103, 0.482]. Those who were uncertain of their husbands’ attitude towards contraceptive use were also 66% less likely to receive FP than clinets who had approvals from their husband AOR = 0.3403 [95% CI; 0.160, 0.722]. The same study from Egypt concluded that women who lack to receive post abortion contraceptives are because of disapprovals from their husband [22].

Clients whom their husband were the main deciders were also 14.9% less likely to receive contraception as compared to the particpants who decide themselves AOR = 0.149 [95% CI; 0.034, 0.650]. A study conducted in Nigeria revealed that those women whose husbands are the only decision makers where associated with low FP utilization [23]. In this study women who discharged with oral pill after they receive abortion care were not followed whether they took or not but were considered as they utilize contraceptive. So, this study finding should be viewed with thiat limitation.

## Conclusions

About one-third of women who received abortion care have failed to receive contraceptives before they leave the facility. Type of health facility, decider on when to have child, knowing women can get pregnant sooner after abortion and husband’s attitude on contraception found to be significant factors for post-abortion contraceptive use. Private facilities should strengthen the post abortion care service. Health care providers should provide counseling on time when fertility-return following abortion before the women left the facility. Partners should be involved during counseling by giving enough time after obtaining the women’s informed consent, to become supportive. Women empowerment through enhancing community’s awareness focusing on own decision making in the family planning utilization should be strengthened.

## Abbreviations

AMHC: Atsede Maryam Health Center
AOR: Adjusted Odds Ratio
CI: Confidence Interval
COR: Crude Odds Ratio
FGAE: Family Guidance Association of Ethiopia
FP: Family Planning
HC: Health Center
HP: Hospital
MCH: Mother and Child Health
MHC: Millenium Health Center
NGO: Non Governmental Organization
PAFP: Post Abortion Family Planning
PAS: Proportional Allocation to Size
SPSS: Statistical Package for Social Science
WHO: World Health Organization
